# Assessing the Learning Curve in Conduction System Pacing Implantation

**DOI:** 10.3390/jcm14248684

**Published:** 2025-12-08

**Authors:** Amato Santoro, Claudia Baiocchi, Maurizio Collantoni, Stefano Lunghetti, Francesco Morrone, Niccolò Manetti, Laura Spaccaterra, Alessia Petrini, Simone Taddeucci, Massimo Fineschi

**Affiliations:** 1 Division of Interventional Cardiology, Azienda Ospedaliera Universitaria Senese, Viale Bracci 11, 53100 Siena, Italy; 2 Department of Biotechnologies, University of Siena, Viale Bracci 11, 53100 Siena, Italyn.manetti@student.unisi.it (N.M.); laura.spaccaterra@student.unisi.it (L.S.);; 3 Division of Cardiology, Azienda USL Toscana Nord Ovest, 55041 Livorno, Italy

**Keywords:** left bundle branch pacing area, his bundle pacing, conduction system pacing

## Abstract

**Background**: Conduction system pacing (CSP) has emerged as an alternative to biventricular pacing (BiVp), but its implantation requires a specific learning curve. Early experience was dominated by His bundle pacing (HBP) with lumenless leads (LLL), whereas the subsequent adoption of left bundle branch area pacing (LBBAP) and the increasing procedural standardization led to a simplification of the technique and greater uniformity in its execution. This study evaluated the learning curve for CSP by analyzing the first consecutive implants of two electrophysiologists (operator 1: OP1 and operator 2: OP2). **Methods:** The first 55 CSP procedures performed by each operator (*n* = 110) were retrospectively analyzed. Implantation and fluoroscopy times were assessed in blocks of five cases. Univariate and multivariable linear regression were used to identify independent predictors of procedural complexity. **Results:** A total of 110 CSP implants (55 per electrophysiologist) were analyzed. Implantation time progressively decreased with experience, reaching a stable plateau after ~45 cases per operator, when inter-operator curves completely overlapped and differences were no longer significant. Fluoroscopy time stabilized earlier and showed no consistent differences between electrophysiologists. In the univariate analysis, longer procedural times were associated with larger left ventricular end-diastolic diameters (LVEDD: r: 0.43; *p* < 0.001), LLL (r: 0.25; *p* < 0.01) and earlier implant numbers (r: 0.36; *p* < 0.001). In the multivariable models, only LVEDD and implant number (IN) remained independent predictors of procedure duration (LVEDD: β: 2.04, *p*: 0.04; and IN: β: 3.26, *p* < 0.04). **Conclusions:** CSP implantation follows a distinct learning curve, with procedure duration stabilizing after approximately 45 cases per operator. Procedural complexity is mainly determined by patient factors (LVEDD) and operator-related factors, whereas differences between LLL and SL reflect historical experience rather than intrinsic technical characteristics.

## 1. Introduction

In recent years, the new technique of conduction tissue pacing has been developed. Initially, it was performed with the implantation of His bundle pacing (HBP), in accordance with the guidelines of the time [1,2]. Over time, it was observed that pacing of the left bundle branch area (LBBAP) was equally effective but with improved electronic parameters; specifically, a better pacing threshold, better sensing, and faster and simpler procedures [3,4]. In subsequent years, a consensus document was published that organized the methods for performing such implants [5]. Conduction system pacing (CSP) originated as a bailout strategy for failures in cardiac resynchronization therapy (CRT) in cases of inadequate lead positioning in the coronary sinus [1,3,4]. The aim of our study was to retrospectively analyze the first 55 implants performed at our center by two electrophysiologists (Operator 1: OP1 and Operator 2: OP2), focusing on the duration of the implants. Furthermore, we identified a learning curve by dividing our observations into groups of five implants for each OP at our single center. As a surrogate parameter to evaluate the learning curve, we used the duration of each procedure and the fluoroscopy time during the procedure.

## 2. Materials and Methods

CSP was performed exclusively as a bailout therapy for BiVp. The bailout strategy was applied in the following circumstances:

- Inadequate coronary sinus venous anatomy (absence of suitable lateral or postero-lateral branches, or only anterior, posterior, or apical veins available);
- Absence of significant intramyocardial conduction delay, either spontaneous or paced (<100 ms) according to our OP lab experiences [6,7,8,9];
- Lack of QRS narrowing during biventricular pacing.

In line with international guidelines and subsequent consensus documents, the first procedures were carried out with HBP; over time, the technique progressively evolved toward LBBAP, which has become our standard approach. With the progressive expansion of indications, CSP was also increasingly performed in patients requiring conventional pacing systems (DDD mode) for advanced atrioventricular block and expected high ventricular pacing burden, as well as in candidates for ablate-and-pace strategies [4,5]. CRT was performed using a back-up lead in the right ventricle (RV) connector. A dual or single chamber pacemaker was used in case of the absence of a back-up lead in the RV. Procedural complications and medium-term CSP-related issues were systematically assessed. Early complications included lead dislodgement, septal perforation, acute threshold rise, pocket complications, and vascular access events. Follow-up evaluations (at 3, 6, and 12 months) assessed pacing thresholds, sensing parameters, R-wave amplitude stability, late septal perforation, loss of capture, infection, and the need for lead revision. All complications were prospectively recorded and categorized by the operator.

### 2.1. CSP with His Bundle Pacing

HBP was performed using the Select Secure pacing system, and a quadripolar diagnostic lead was utilized to locate and target the His bundle area. Following this, both steerable and fixed delivery were introduced into the RV. The devices used for His bundle pacing included Medtronic models, such as the Select Secure (C3155 HIS) and steerable delivery (model C304 HIS). The lead was placed to detect the His bundle signal during pacing tests, and His bundle capture was confirmed. The lead was screwed in when either selective or non-selective His bundle capture was achieved. Sensing and threshold tests were repeated to ensure that the electrical parameters were within acceptable limits (capture threshold < 2 V at 1 ms). Selective or non-selective His bundle capture was defined according to the consensus criteria established by a collaborative working group [2]. In cases where the threshold was high (>2 V at 1 ms) with HBP, LBBAP was performed as an alternative. LBBAP was carried out as previously described, and capture was confirmed using the criteria provided in the literature [2,3].

### 2.2. CSP with Left Bundle Branch Pacing

The delivery was advanced into the right ventricle using the over-the-wire technique with a hydrophilic guide. Alternatively, the lead was advanced into the right ventricle and then placed in the septal region. Another approach involved placing the hydrophilic guide or lead in the right outflow tract, advancing the delivery to the septal region of the right outflow tract, and then retracting it to place the delivery against the septum. A contrast medium injection was performed according to the EP preference. Pacing was carried out in the septal region until a W pattern in V1 was obtained, along with discordance in the inferior leads or negative concordance in the inferior leads. The lead was then advanced until there was a transition from the paced left bundle branch block pattern to the paced right bundle branch block pattern morphology, while simultaneously achieving the shortest interval between the stimulus and the peak R-wave in V6 with minimal output. Once the appropriate regions were identified, the screw was advanced depending on the type of lead used. When r-R’ was observed in V1 and blocked ectopic beats were present, the pacing parameters were checked. For each implant, LVAT and R’ in V1-RV6 were measured, in accordance with the EHRA consensus document^5^. If the parameters were unsatisfactory, the lead was repositioned or further advanced. LBBAP was defined as capturing either the proximal left bundle or the septal left bundle with an intermediate QRS axis (showing discordance in DII/DIII), or capturing the anterior or posterior fascicles with a superior (both DII and DIII negative) or inferior (both DII and DIII positive) QRS axis, respectively. The procedure was considered successful when the EHRA consensus document criteria were met, particularly:

- Achievement of a stable and selective or non-selective LBBAP/His capture;
- R-R’ in V1 and blocked ectopic beats were present;
- Appropriate 12-lead ECG pattern consistent with CSP physiology;
- LVAT measurement confirming physiological activation, according to the EHRA document [5];
- Acceptable pacing thresholds and sensing parameters.

#### 2.2.1. Clinical Data

Baseline demographic, clinical, electrocardiographic, and echocardiographic data of included patients were prospectively collected and reported in a dedicated database. The same data were collected at the 1-year follow-up as well. Echocardiograms were performed using a Vivid^TM^ iQ ultrasound system (GE Healthcare, Chicago, IL, USA), equipped with an adult transthoracic 1.5–4.0 MHz probe in accordance with the recommendations of the American Society of Echocardiography/European Association of Cardiovascular Imaging [10]. LV ejection fraction (LVEF) was obtained using the biplane modified Simpson’s method from the apical four- and two-chamber views.

#### 2.2.2. Statistical Analysis

Continuous variables were expressed as mean ± standard deviation, and categorical variables as counts and percentages. Comparisons between operators across blocks of five implants were performed using Welch’s *t*-test for independent samples, with the Mann–Whitney U test applied as a sensitivity analysis. The association between the progressive implant number (IN) and procedural/fluoroscopy time was assessed using the Pearson correlation coefficient (r), with significance tested by a two-tailed *t*-test. Determinants of implantation and fluoroscopy times were investigated using linear regression analyses. Univariate analyses were performed first, and variables with *p* < 0.10 were entered into multivariable ordinary least squares (OLS) regression models. Multiple linear regression analysis was performed to identify independent predictors of procedural and fluoroscopy duration. Variables showing a *p*-value < 0.10 in univariate analysis were entered into the multivariate model. For each predictor, statistical significance was assessed using the *t*-test for regression coefficients (β), while the global fit of the model was evaluated with the ANOVA F-test. Regression coefficients (β), 95% confidence intervals, *p*-values, and model R^2^ are reported. All statistical analyses were performed using IBM SPSS Statistics, version 20.0 (IBM Corp., Armonk, NY, USA).

## 3. Results

The characteristics of the study population were similar. No differences were observed between the patients undergoing CSP implantation in the OP1 and OP2 groups, as reported in Table 1. Approximately one-third of the patients were female. CSP implants were performed as a bailout strategy in cases of failure or inadequate CRT, as described in the Section 2. The average post-implant parameters were similar for both the duration of the paced QRS (pQRS) and the HV and LVAT parameters, respectively, for HBP and LBBAP, as shown in Table 2. The pQRS length was not statistically significant between OP and was therefore homogeneous. The clinical indication for CSP implantation was systematically recorded and is detailed in Table 2, which distinguishes bailout CSP during CRT attempts, primary implants for atrioventricular block (AVB), and ablate-and-pace (A/P) indications. This classification was used to contextualize procedural duration, complexity, and pacing metrics for each operator.

### 3.1. Procedure Duration

**Figure 1:**
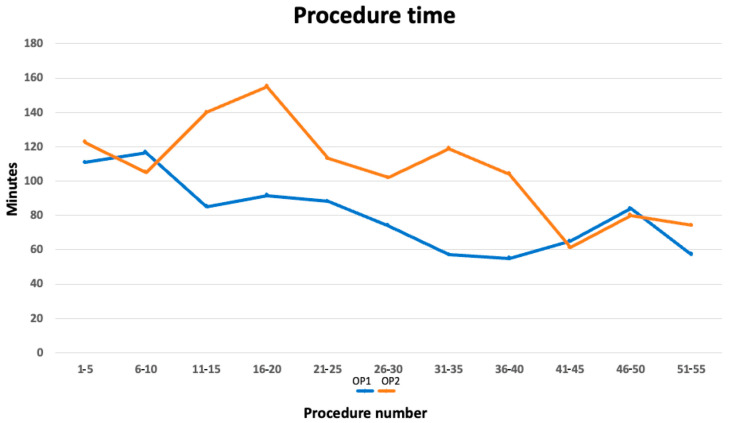
The graph shows the duration times for performing a CSP. The two curves represent two electrophysiologists. After the 40th case, the duration curves overlap.

The duration of the procedure showed a decreasing trend with the increasing number of procedures performed, as reported in Figure 1. A difference in procedure duration was observed until the completion of approximately 45 CSP implants. When the learning curve was analyzed in blocks of five consecutive implants per operator, no significant difference in implantation time was observed during the first two blocks, as reported in Table 3. A marked difference emerged in blocks 3 and 4, with OP2 requiring longer procedures (borderline significance at block 3, *p* = 0.05; block 4, *p* = 0.08). Between blocks 5 and 7, the difference persisted but did not reach statistical significance. A further significant divergence was observed at block 8 (*p* = 0.003). From block 9 onward (corresponding to approximately the 45th implant for each OP), implantation times became consistently comparable between operators, with no further statistically or clinically relevant differences. This finding indicates that after about 45 procedures per operator, performance stabilizes and the learning curve plateaus. Table 3 provides a detailed overview of the materials used by the operators. Notably, there was an initial use of lumenless lead (LLL) and HBP, in accordance with the indications from the earliest studies on conduction tissue pacing. Over time, and with the increase in the number of implants, there was a marked reduction in the use of HBP in favor of LBBAP and the use of a stylet-driven lead (SL).

### 3.2. Fluoroscopy Time

**Figure 2:**
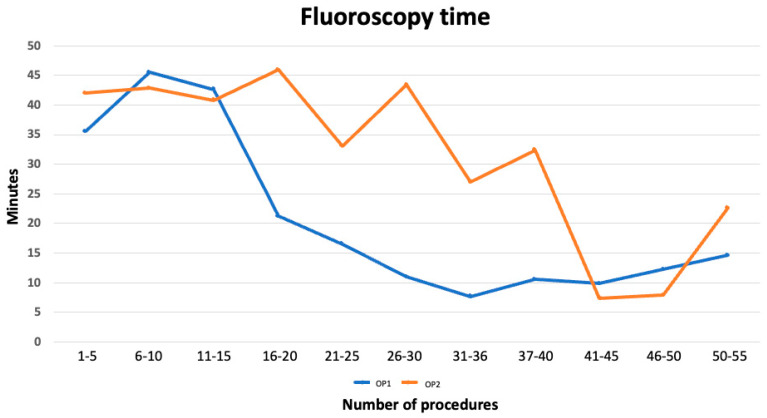
The graph shows the fluoroscopy times for performing a CSP. The two curves represent two electrophysiologists.

Analysis of fluoroscopy time across blocks of five consecutive procedures per operator did not reveal statistically significant differences. During blocks 4 to 8, OP2 tended to require longer fluoroscopy times, although the differences did not reach significance, as reported in Table 4 (*p*-values between 0.13 and 0.26 with Welch’s *t*-test; Mann–Whitney U test, *p* ~0.10). From block 9 onward, mean fluoroscopy times were consistently comparable between the two OPs, with no clinically or statistically relevant differences. This suggests that, unlike implantation time, fluoroscopy performance between OPs was substantially aligned from the early phases of the learning curve, with no clear plateau effect detectable as reported in Figure 2.

### 3.3. Univariate and Multivariate Analysis

#### 3.3.1. Implantation Time

**Figure 3:**
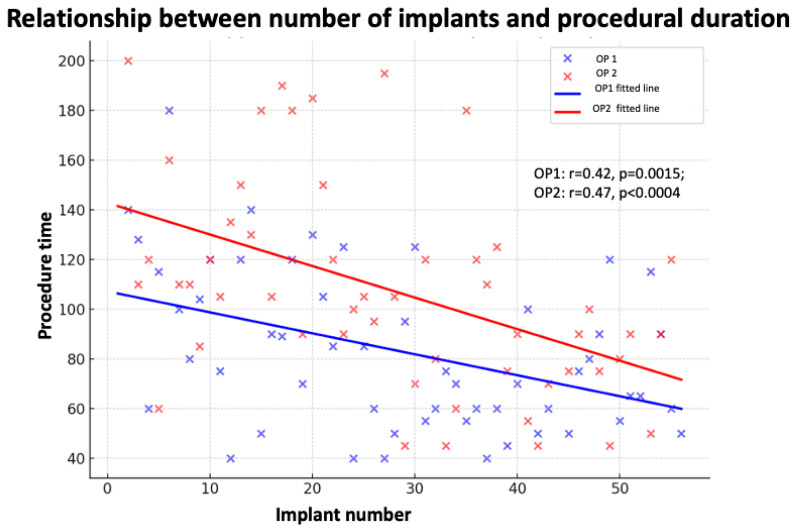
Relationship between the progressive number of implants and procedural duration for the two electrophysiologists (OP1 and OP2). A significant inverse correlation was observed, consistent with the presence of a learning curve. For OP1: r = −0.42, *p* = 0.0015; for OP2: r = −0.47, *p* = 0.0004. A negative correlation coefficient indicates that the greater the number of procedures performed, the shorter the mean implantation time.

The univariate and multivariate analyses are shown in Table 5. In the univariate analysis, both left ventricular end-diastolic diameters (LVEDD) (r: 0.43; *p* < 0.001) and progressive IN (r: 0.36; *p*< 0.001) were significantly associated with procedure duration, as reported in Figure 3. In addition, the use of LLL was associated with longer implantation times compared with SL (r: 0.25; *p* < 0.01). No significant associations were observed for QRS morphology (LBBB), device type (three chamber PM vs. dual or single chamber), or pacing site (HBP vs. LBBAP). In the multivariate model, LVEDD and IN remained independent predictors, while the effect of the lead system was no longer significant for both OPs. Implantation time was inversely correlated with the progressive number of procedures for both OPs, confirming the presence of a learning curve. For OP1, the correlation was r = −0.42 (*p* = 0.0015), while for OP2 it was r = −0.47 (*p* = 0.0004). These negative correlation coefficients indicate that the higher the number of implants performed, the shorter the mean procedural duration. As the number of procedures increased, a gradual reduction in procedure duration was observed, as reported in Figure 3 for each OP. The duration of the procedure is inversely correlated with the number of interventions performed by both OP.

#### 3.3.2. Fluoroscopy Time

In the univariate analysis, LVEDD (r: 0.37, *p* < 0.001), IN (r: 0.28, *p* < 0.001), and LLL (r: 0.26, *p* < 0.05) were significantly associated with fluoroscopy time. No significant association was found for ECG pattern, EF, device type, or pacing site. In the multivariate model, LVEDD remained an independent predictor. During the initial phase of the experience, only LLL were used, as these represented the sole CSP leads available in our center. With the subsequent introduction of SL, their use progressively increased and became comparable across later procedures. The natural evolution in the availability of implant tools included not only different lead types but also a broader selection of delivery sheaths. During the study period, all CSP materials from the different manufacturers were gradually introduced and adopted. In the earliest phase, only LLL leads were available and therefore used exclusively; as additional tools became commercially accessible, SL leads and the full range of delivery systems from all companies were progressively incorporated into routine practice. Both LBBPa and His bundle pacing were performed throughout the experience. Detailed per-procedure information (specific lead model, manufacturer, delivery tools, and pacing modality) is provided in Supplementary Table S1. The higher number of implants and the refinement of the technique led to the use of both systems and catheters without any difference between them or between OP. The introduction of new implantation tools during the study period reflects the natural evolution of CSP technology. Early procedures relied exclusively on LLL leads, while later cases incorporated SDL leads in comparable proportion, together with a wider selection of delivery systems. Although these changes may theoretically act as procedural confounders, the progressive reduction in procedure duration, fluoroscopy exposure, and complication rates persisted independently of the specific hardware used. Importantly, once the operators had reached an adequate level of experience, all lead types and delivery systems were handled with similar ease, and procedural performance improved regardless of whether LLL or SL were used, indicating that the learning curve is primarily operator-dependent rather than tool-dependent. Full technical details are provided in Supplementary Table S1.

#### 3.3.3. Complications

The second implant performed by OP1 resulted in a small interventricular septal defect, which was not hemodynamically significant. The third implant performed by OP2 using an LLL developed a self-limiting pericardial effusion. The fourth and sixth implants with SL (Ingevity Boston Scientific and Tendril Abbott, respectively) resulted in lead coil fracture and lead fracture, without patient complications, and the procedure was completed with an additional lead [11]. Both complications were performed by OP1. The only complication performed by OP2 using an SL was coil spiralization, related to failure of the outer coil (Ingevity + Boston Scientific) during the first implant with an SL. In this case, the lead was repositioned, and an adequate conduction tissue pacing implant was performed.

### 3.4. Figures, Tables, and Schemes

Medium-term follow-up confirmed the safety and stability of CSP implants performed throughout the learning curve. Pacing thresholds and sensing parameters remained stable, and no late septal perforation or clinically relevant deterioration of CSP performance was observed. These findings provide an additional quality measure supporting the procedural feasibility and safety of CSP even during the early phases of operator training.

## 4. Discussion

CSP implantation is characterized by a gradual learning curve. At our center, the technique was introduced in 2019, in the absence of proctorship or external expertise, at a time when CSP was just beginning to spread and was not yet routine practice in many electrophysiology laboratories [12,13,14,15,16]. As expected, the increasing number of procedures was the primary determinant of a faster and more efficient implant. Initially, the use of LLL represented the main technical challenge, requiring a new implantation strategy. Early experience was also marked by isolated complications, such as a small interventricular septal defect during the second implant, which had no hemodynamic significance. With the progressive adoption of both LLL and SL systems, and the subsequent introduction of LBBAP, procedure times progressively decreased [15,16,17]. In our experience, the availability of multiple systems allowed the optimal selection of the most suitable lead for each patient and indication. Our analysis showed that LVEDD and IN were the main independent determinants of procedural complexity. LVEDD consistently predicted longer implantation and fluoroscopy times, confirming its role as a marker of procedural difficulty. LVEDD, which may reflect the degree of LV dilatation, emerged as an independent predictor of implantation and fluoroscopy times. Larger ventricular size is likely to increase procedural complexity by making lead positioning and septal penetration more challenging, thereby prolonging CSP implantation. Conversely, the progressive IN captured the learning curve effect, with shorter procedures as experience accumulated. Lead type deserves specific mention. In the univariate analysis, LLL was associated with longer implantation times. This finding is best explained by the historical context: early CSP procedures were exclusively His bundle pacing with the only LLL available, performed when EP experience was limited and technical uncertainties were high. At the beginning of CSP in 2019, LLL was the only system available, and its implantation technique was completely different from conventional SL. As it was a novel approach, with limited dissemination, absence of structured proctoring, and scarce guidance on maneuverability, early procedures were associated with longer implantation times. With the subsequent publication of consensus documents and increasing OP familiarity, the usability of LLL became well established^18^. Consequently, lead type no longer impacts implantation duration in current practice. As the technique became consolidated and consensus documents provided clear recommendations, differences between LLL and SL systems disappeared. Stylet-driven leads were already well known and widely used in conventional pacing [16,17,18]. For this reason, they appeared more familiar to operators and seemed to be associated with a shorter learning curve for CSP compared with LLL, although in our opinion, this difference mainly reflected historical context rather than intrinsic technical advantages. For physicians starting CSP today, with standardized methods and consolidated knowledge, such differences would likely not emerge at all. In summary, CSP procedural complexity is primarily influenced by LVEDD and OP experience. Apparent associations with lead type reflect the early learning curve rather than intrinsic differences between delivery systems.

### Study Limitations

The main limitation of this study is the small sample size and the comparison of only two OPs. Another limitation is that the study was conducted at a single center rather than being multicenter. The execution of CSP today is undoubtedly more straightforward compared with our experience, as we began in the absence of a consensus document, which was published later, and with limited reports, but not numerous cases described in the literature. Starting with only the LLL technology available and using a “new” lead for the OP represents an additional limitation of this observational study.

## 5. Conclusions

In our experience, the main determinants of implantation and fluoroscopy time were the LVEDD and the progressive IN. Procedural duration progressively decreased with experience, and after approximately the 45th case, the differences between OPs became negligible, with overlapping implantation and fluoroscopy times. The apparent association between LLL and longer procedures was limited to the earliest phase of the learning curve, when His bundle pacing was initiated exclusively with LLL. With the subsequent adoption of SL, the diffusion of LBBAP, and the availability of consensus guidelines, procedural times equalized across lead types. Taken together, these findings indicate that a dilated heart and IN are the true independent determinants of CSP procedural complexity, while lead type reflects the historical context of the learning curve rather than intrinsic technical differences.

## Figures and Tables

**Table 1:** Population characteristics.

	Female (*n*. %)	AGE (m/SD)	BSA (m/SD)	RWT m/SD	LVEDD (m/SD)	EF % (m/SD)
**OP 1**	18/32.7	75.5 ± 8.1	0.9 ± 0.1	0.42 ± 0.13	59.3 ± 9.4	41.7 ± 15.3
**OP 2**	15/27.3	72.9 ± 25.9	1 ± 0.2	0.41 ± 0.2	57.4 ± 26.3	40.2 ± 22.3

BSA: body surface area; RWT: relative wall thickness; LVEDD: left ventricular diastolic diameter; EF: ejection fraction.

**Table 2:** CSP indication and CSP parameters.

	Bailout (*n*./%)	AVB (*n*./%)	A/P (*n*./%)	pQRS ms (m/SD)	LVAT ms (m/SD)	HV ms (m/SD)
**OP 1**	41/74.5	7/12.7	7/12.7	108.2 ± 13.2	69.9 ± 10.1	43.7 ± 15.4
**OP 2**	43/78.2	7/12.7	5/9.1	106.2 ± 35.6	68.5 ± 34.7	42.2 ± 22.5

AVB: atrio-ventricular block; A/P: ablate and pace; pQRS: paced QRS duration; LVAT: left ventricular activation time; HV: His-Ventricular interval.

**Table 3:** Procedure time.

Block	1	2	3	4	5	6	7	8	9	10	11
*n*.cases	1–5	6–10	11–15	16–20	21–25	26–30	31–35	36–40	41–45	46–50	51–55
**OP 1 m/SD**	110.7 ± 43.5	116.5 ± 18.3	85 ± 43.2 *	91.5 ± 24.6 *	88 ± 36.5	74 ± 23.3 *	57 ± 31.9 *	55 ± 10.9 *	65 ± 21.8	84 ± 17.1	57.2 ± 26.8
**OP 2 m/SD**	122.7 ± 32.1	105 ± 31.4	140 ± 16.8	155 ± 39.4	113 ± 40.8	102 ± 42.1	119 ± 31.2	104 ± 49.2	61.2 ± 29.5	80 ± 18.7	74.25 ± 45.7

* *p* < 0.05 between OP1 and OP2.

**Table 4:** Fluoroscopy time.

Block	1	2	3	4	5	6	7	8	9	10	11
*n*.cases	1–5	6–10	11–15	16–20	21–25	26–30	31–35	36–40	41–45	46–50	51–55
**OP 1 m/SD**	35.5 ± 22.1	45.5 ± 27.7	42.6 ± 8.7	21.2 ± 3.9 *	16.5 ± 6.4 *	10.9 ± 8.7 *	7.7 ± 11.4 *	10.5 ± 5.6 *	9.8 ± 7.9	12.3 ± 5.9	14.6 ± 2.2
**OP 2 m/SD**	42 ± 18.5	42.8 ± 26.2	40.8 ± 18.3	45.9 ± 18.3	33.1 ± 26.8	43.4 ± 6.4	27 ± 9.4	32.4 ± 18.5	7.4 ± 3.6	7.9 ± 3.2	22.6 ± 9.4

* *p* < 0.05 between OP1 and OP2.

**Table 5:** Univariate and multivariate analysis.

Implant Duration	Predictor	r	Univariate β (95% CI)	*p* (uni)	Multivariate β (95% CI)	*p* (Multi)
	LBBB ECG	0.01	8.78 (−6.83 to 24.39)	0.267	7.99 (−39.81 to 55.78)	0.725
	LVEDD (mm)	0.43	0.86 (0.43 to 1.29)	<0.001	2.04 (−0.10 to 4.18)	0.04
	EF	−0.07	−0.19 (−0.47 to 0.10)	0.194	0.40 (−1.18 to 1.97)	0.598
	CRT/PM	0.08	2.76 (−12.38 to 17.90)	0.719	−14.92 (−62.29 to 32.46)	0.510
	LLL	0.25	23.52 (8.08 to 38.96)	0.003	14.09 (−38.34 to 66.52)	0.573
	HBP/LBBPa	0.09	25.25 (−7.98 to 58.48)	0.131	13.54 (−78.66 to 105.74)	0.757
	Implant number	0.36	−1.07 (−1.51 to −0.63)	<0.001	3.26 (−1.57 to 6.49)	0.04
**Fluoroscopy time**	**Predictor**	**r**	**Univariate β (95% CI)**	***p* (uni)**	**Multivariate β (95% CI)**	***p* (multi)**
	LBBB ECG	0.2	11.58 (0.71 to 22.45)	0.037	0.55 (−35.82 to 36.93)	0.974
	LVEDD (mm)	0.37	0.39 (0.10 to 0.68)	<0.001	2.01 (−0.37 to 3.39)	0.04
	EF	−0.07	0.01 (−0.19 to 0.21)	0.927	−0.06 (−1.24 to 1.12)	0.917
	CRT/PM	0.08	6.68 (−3.78 to 17.14)	0.207	7.63 (−22.09 to 37.34)	0.584
	LLL	0.26	11.55 (1.08 to 22.01)	0.031	−9.44 (−43.23 to 24.35)	0.551
	HBP/LBBPa	0.1	5.90 (−14.38 to 26.18)	0.553	1.04 (−56.45 to 58.52)	0.969
	Implant number	0.28	−0.60 (−0.89 to −0.30)	<0.001	−0.23 (−2.92 to 2.46)	0.856

Abbreviations: LBBB: left bundle branch; CRT: cardiac resynchronization therapy; PM: DDD or VVI pacemaker; LVEDD: interventricular conduction delay; EF: ejection fraction; LLL: lumenless; PM: pacemaker. Implant number is progressive per operator (1–55).

## Data Availability

Anonymized datasets are available from the corresponding author upon reasonable request and with institutional permission.

## Supplementary Materials

The following supporting information can be downloaded at: https://www.mdpi.com/article/10.3390/jcm14248684/s1, Table S1: Tools and specific typologies of CSPs performed.
