# Supplementary material for: Assessing the Learning Curve in Conduction System Pacing Implantation
Source: J Clin Med. 2025 Dec 8;14(24):8684. doi: 10.3390/jcm14248684 (PMC12734241; doi:10.3390/jcm14248684)
Supplement: Supplementary file 1 [file jcm-14-08684-s001.zip › jcm-3995844-supplementary.pdf]

Supplemental material Table

Tools and specific typology of CSP performed.

	LL	SL	LL	SL	LL	SL	LL	SL	LL	SL	LL	SL	LL	SL	LL	SL	LL	SL	LL	SL	LL	SL
N.	**1-5**		**6-10**		**11-15**		**16-20**		**21-25**		**26-30**		**31-35**		**36-40**		**41-45**		**46-50**		**51-55**	
EP 1 (number of cases)	5	0	4	1	2	3	2	3	5	0	1	4	0	5	1	4	2	3	3	2	1	4
n. LBBPa	0	0	3	1	2	1	2	3	4	0	1	4	0	5	1	4	2	2	3	2	1	3
n. His	5	0	1	0	0	2	0	0	1	0	0	0	0	0	0	0	0	1	0	0	0	1
Delivery (n.)	13	0	5	3	3	6	2	4	6	0	1	4	0	5	1	4	2	3	3	3	1	4
Leads (n.)	6	0	6	2	3	5	2	4	6	0	1	4	0	5	1	4	2	3	3	3	1	4
Positioning (n.)	16	0	9	4	4	6	3	5	7	0	2	6	0	6	1	5	3	3	3	2	1	4
EP 2 (number of cases)	5	0	5	0	4	1	4	1	4	1	3	2	5	0	1	4	3	2	1	4	1	4
n. LBBPa	0	0	0	0	0	0	3	1	4	0	3	2	4	0	1	4	3	2	1	4	0	4
n. His	5	0	5	0	4	1	1	0	0	1	0	0	1	0	0	0	0	0	0	0	1	0
Delivery (n.)	9	0	6	0	6	3	4	2	4	1	3	2	6	0	2	5	3	2	2	4	0	5
Leads (n.)	6	0	5	0	5	2	4	2	4	2	3	2	5	0	1	4	3	2	1	4	0	5
Positioning (n.)	12	0	7	0	8	3	5	3	5	2	3	3	6	0	1	4	3	2	2	4	0	6

LL: lumenless lead. SL: stylet driven lead.
Total LL: 62; Total SL: 48. SL: EP 1: 29. EP 2: 19 . LL: EP 1: 26. EP 2: 36.
TOTAL LEAD POSITIONED:
Medtronic lumenless Select Seure 3830 pacing lead (n. 62)
Abbott Tendril STS, pacing lead, 55 cm (n. 6)
Boston Scientific, Ingevity, pacing lead 58 cm (n.4). Ingevity + pacing lead, 58 cm (n.23)
Biotronik Solia S 60 pacing lead, 60 cm (n. 11)
Microport Vega 58 cm (n. 4).
